# Novel Non-Invasive Quantification and Imaging of Eumelanin and DHICA Subunit in Skin Lesions by Raman Spectroscopy and MCR Algorithm: Improving Dysplastic Nevi Diagnosis

**DOI:** 10.3390/cancers14041056

**Published:** 2022-02-18

**Authors:** José Javier Ruiz, Monica Marro, Ismael Galván, José Bernabeu-Wittel, Julián Conejo-Mir, Teresa Zulueta-Dorado, Ana Belén Guisado-Gil, Pablo Loza-Álvarez

**Affiliations:** 1 ICFO-Institut de Ciencies Fotoniques, The Barcelona Institute of Science and Technology, Castelldefels, 08860 Barcelona, Spain; jose-javier.ruiz@icfo.eu; 2 Department of Evolutionary Ecology, National Museum of Natural Sciences, CSIC, 28006 Madrid, Spain; galvan@mncn.csic.es; 3 Department of Dermatology, University Hospital Virgen del Rocio, 41013 Sevilla, Spain; jose.bernabeu.sspa@juntadeandalucia.es (J.B.-W.); jsconejomir@us.es (J.C.-M.); teresa.zulueta.sspa@juntadeandalucia.es (T.Z.-D.); anab.guisado.sspa@juntadeandalucia.es (A.B.G.-G.)

**Keywords:** skin neoplasms, melanoma, dysplastic nevus syndrome, eumelanin, Raman spectroscopy analysis, multivariate analysis, reactive oxygen species

## Abstract

**Simple Summary:**

The diagnosis of dysplastic nevi is a dermatological challenge since it is an intermediate lesion between benign and malignant tumors. Currently, clinical diagnosis relies on biopsies and subsequent histopathologic examinations, which are invasive, expensive, time-consuming and subjective. Accordingly, in this work, we evaluate the potential of Raman spectroscopy, coupled with multivariate analytical methods, to non-invasively diagnose skin tumor biopsies by characterizing their pigment composition. We show an innovative methodology for a non-invasive quantification and localization of the eumelanin pigment and its DHICA subunit in skin lesions, which represents a further step to analyze the pigment content compared to the established invasive technique HPLC. Furthermore, we report for the first time that the DHICA content in dysplastic lesions is lower than in benign and malignant ones. This leads to the accurate classification of dysplastic lesions with 94.1% sensitivity and 100% specificity in an objective, cost-effective, non-invasive and rapid way.

**Abstract:**

Malignant melanoma (MM) is the most aggressive form of skin cancer, and around 30% of them may develop from pre-existing dysplastic nevi (DN). Diagnosis of DN is a relevant clinical challenge, as these are intermediate lesions between benign and malignant tumors, and, up to date, few studies have focused on their diagnosis. In this study, the accuracy of Raman spectroscopy (RS) is assessed, together with multivariate analysis (MA), to classify 44 biopsies of MM, DN and compound nevus (CN) tumors. For this, we implement a novel methodology to non-invasively quantify and localize the eumelanin pigment, considered as a tumoral biomarker, by means of RS imaging coupled with the Multivariate Curve Resolution-Alternative Least Squares (MCR-ALS) algorithm. This represents a step forward with respect to the currently established technique for melanin analysis, High-Performance Liquid Chromatography (HPLC), which is invasive and cannot provide information about the spatial distribution of molecules. For the first time, we show that the 5, 6-dihydroxyindole (DHI) to 5,6-dihydroxyindole-2-carboxylic acid (DHICA) ratio is higher in DN than in MM and CN lesions. These differences in chemical composition are used by the Partial Least Squares-Discriminant Analysis (PLS-DA) algorithm to identify DN lesions in an efficient, non-invasive, fast, objective and cost-effective method, with sensitivity and specificity of 100% and 94.1%, respectively.

## 1. Introduction

Malignant melanoma (MM) is the most aggressive form of skin cancer, with an increasing incidence rate worldwide. Although its incidence is much lower than that of non-melanoma skin cancer (NMSC) lesions, it causes the majority (approximately 90%) of skin cancer-related deaths [1]. MM can be completely curable if it is detected at an early stage; however, they have a high metastatic capability, and a particularly effective treatment of metastatic melanoma is not currently available [2]. Therefore, significant efforts are addressed on early detection tools that allow a fast clinical diagnosis, which may be decisive for patient survival.

Dysplastic nevus (DN) is a lesion type whose definition is a matter of debate, as there is no consensus on terminology among dermatologists or dermatopathologists [3]. One of the reasons is because, in terms of clinical, microscopic, morphological and genomic aspects, these are intermediate lesions between benign nevi and melanomas [4]. For these reasons, DN are the most difficult lesions to be identified, to such an extent that clinical picture, dermatocospy, molecular or genetic methods are often not able to distinguish DN and MM lesions [5]. Despite this, DN is recognized as a histologic entity whose biological relevance is that their appearance can be considered as a marker of melanoma risk [3,6,7,8]; for instance, the risk of suffering from melanoma increases 10-fold in people with ≥5 DN [9], and the risk is also higher with the presence of nevi with a high grade of atypia [8]. However, it is still debated if DN are precursors of melanomas [3], as only a small number of them are reported to evolve to malignancy [9,10]. One of the reasons why this question has not been answered yet is because it is not possible to identify a lesion as DN without biopsy, and this prevents the long-term study of its evolution [3]. On top of that, the lack of unanimous terminology has affected the diagnosis of DN since it is usually based on subjective histological criteria. In general, good intra-observer reproducibility but poorer inter-observer correlation was found [3] and, concretely, the worse agreement was reported while grading DN lesions into “mild”, “moderate” and “severe” categories [3,8]. Consequently, there are no well-established guidelines to aid clinicians with the excision of these lesions [11] since they usually depend on their grade of atypia. Although there is widespread agreement that DN with a severe grade of atypia should be re-excised with appropriate surgical margins, there is disagreement managing mildly or moderate DN, and the decision to re-excise depends on the involvement of surgical margins during initial biopsy [11]. More recently, it was suggested that DN with mild and moderate grades should be safely observed rather than excised [12]. Considering all this, a high necessity currently exists for developing tools able to provide an accurate clinical identification of DN lesions and to avoid the performance of biopsies. This would also allow us to study their evolution after their in vivo diagnosis and answer the question of whether they really evolve to melanomas.

Detection and classification of skin tumors usually rely on the visual inspection and clinical history of patients. Although the skin is an accessible site for inspection, the diagnosis of skin tumors is complex because benign lesions may visually resemble malign ones. Accordingly, a precise clinical diagnosis relies on biopsies and subsequent histopathologic examinations, which represents a challenge for clinicians to decide whether and when to biopsy [13]. Previous studies have assessed the accuracy of MM diagnosis by general practitioners (GPs) and dermatologists. For GPs, sensitivity and specificity values of 62% and 63%, respectively, were reported [14]. For dermatologists, 81–90% sensitivities and 59–60% specificities were measured depending on their experience [14]. These specificity values imply a false positive rate around 40%, which means a high percentage of negative biopsies. That is, biopsies that are unnecessarily performed and imply an invasive risk for patients and an economic loss for hospitals. For these reasons, there is a need for fast, non-invasive and automated tools that help to provide objective clinical decisions [15].

Several optical diagnosis methods were developed, such as total body photography, digital dermoscopy, reflectance confocal microscopy or stepwise two-photon fluorescence microscopy, but none of them are able to provide a final diagnosis or to absolutely replace the histopathological examination [1]. Thus, the necessity of a definite and accurate non-invasive method for skin cancer diagnosis is still pending.

Raman spectroscopy (RS) is a non-invasive, fast and label-free technique, which is based on the inelastic scattering of monochromatic radiation that provides specific vibrational fingerprints of molecules. Hence, it can be implemented in order to obtain privileged information about the chemical content present in biological samples [16]. In the past decade, RS was demonstrated to be a powerful technique with the help of multivariate data analysis (MA), which is usually necessary to interpret and extract the complex information encoded in Raman spectra [17]. Hence, we propose RS in combination with MA as a promising and appropriate method to classify skin tumors, especially to distinguish DN from MM lesions. The methodology proposed here presents two advantages: (a) It is non-destructive because RS analysis does not damage the tissue under investigation or require the addition of labels, and therefore, the biopsies extracted could be analyzed by RS and with complementary techniques such as histopathology or fluorescence microscopy after. (b) Additionally, the study presented in this paper could be potentially translated to in vivo measurements, being non-invasive. The use of fiber optic Raman probes was demonstrated to be a good approach to perform in vivo Raman measurements of the skin [18,19,20]. We believe that the methodology that we present, including advanced MA approaches, has the potential to allow successful results on in vivo implementation in the skin.

The efficiency of RS and MA to discriminate between benign and malignant skin lesions was previously shown; however, very few studies focused on the diagnosis of dysplastic nevi. Searching in the literature, one finds that most of the studies are aimed at the diagnosis of MM, basal cell carcinoma (BCC) and squamous cell carcinoma (SCC), as these are the three most common types of skin cancer [20,21]. Additionally, usually, DN lesions are not considered individually but included with other types of benign tumors in the same group or class. For instance, one of the most comprehensive studies to date of skin tumor diagnosis by RS is the one of Lui et al. [19], in which they classified 518 validated lesions of 10 types of skin tumors measured in vivo. In this study, they classified cancerous and precancerous lesions (MM, BCC, SCC and actinic keratosis) from benign conditions (DN, compound nevi, blue, intradermal, junctional nevi and seborrheic keratosis). That is, they included DN lesions in a class of considered benign lesions. Additionally, in a more recent study [21], they classified 731 lesions of 16 types of and performed a similar group dichotomy to the previous study by grouping different benign lesions all together and including DN. They also showed that the classification accuracy is improved if patient demographic information (gender, skin type, lesion location and age) is included. The classification accuracies obtained in these studies are gathered in Table S1. Additionally, previous studies have labelled skin lesions as pigmented nevi [22] or non-melanoma pigmented lesions [23], without a more detailed specification of which is the lesion type, such as compound or dysplastic nevi, for instance, and more examples can be found in the table of references of the review [20]. Although DN lesions are usually included in the group of benign lesions, due to the lack of consensus whether DN lesions are benign or not, Santos et al. [24] decided to exclude DN samples before calibrating a predictive model to classify MM from common nevi (dermal, compound, intradermal, junctional and blue nevi). After that, they predicted the tumor type of 46 DN samples and, interestingly, 34 were classified as MM and 12 as benign lesions. In other words, their predictive model detected a shift towards a malignant behavior in DN samples, which contradicts the assumption that DN lesions should be grouped as benign ones. Therefore, DN lesions should be considered separately from better defined benign lesions. Finally, it is remarkable the work performed by Feng et al. [25], who developed an inverse biophysical model with Raman components obtained in situ from different skin regions: collagen, elastin, triolein, nucleus, keratin, ceramide, water and melanin, to later apply it to in vivo skin cancer screening data. Later, they used this model to classify skin lesions and discriminated MM from DN with 95% sensitivity and 94% specificity [26] and showed that the most important components in this discrimination are collagen and triolein. Considering all of this, RS is a promising technique in the field of skin cancer diagnosis, but little attention is paid to the specific non-invasive diagnosis of DN lesions. Concretely, in this study, we characterize the eumelanin content of MM, DN and compound nevi (CN) lesions to later discriminate them, with special attention to the DN type.

Although RS was explored in the past to classify skin tumors, this technique was not fully exploited to extract the molecular spatial distribution of the pigments present in skin lesions. Up to date, the standard method to quantify pigments in the skin is the High-Performance Liquid Chromatography (HPLC) method, which is a chemical degradative method by which the sample is homogenized, and the pigment localization is not possible [27,28]. To achieve a non-invasive quantification and localization, the Multivariate Curve Resolution-Alternating Least Squares (MCR-ALS) method is proposed, as it enables us to obtain the concentration and spatial distribution of molecular components in tissue samples from complex Raman spectral images [17,29,30]. Specifically, it was used in the past to study the different skin constituents in tissue sections [25] or the permeation of retinol in the skin [31]. However, RS coupled with MCR-ALS were not exploited to investigate the concentration and distribution of pigments in skin pathological issues. Therefore, here we propose the use of MCR-ALS to study in depth the pigment content and localization in skin lesions.

Nowadays, one of the main challenges is to distinguish between DN and MM. In a previous study [32], the reported sensitivity and specificity of dermatologists to diagnose DN using the standard ABCD rule were 84.4% and 74.5%, respectively. In Table S1, the different reported accuracies of GPs, dermatologists and different proposed methods to distinguish the skin lesions considered in this study (MM, DN and CN) are summarized. To date, few studies have focused on the distinction of DN, even fewer in a non-invasive way. Then, the aim of this study is thus to assess the accuracy of RS and MA to classify skin tumor biopsies of malignant melanoma (MM), compound nevi (CN) and dysplastic nevi (DN), with special attention to the latter. CN are fully benign lesions without the ability to metastasize, and it is the type of benign melanocytic found more frequently. Therefore, CN can be used as a control.

To test the potential of RS to diagnose skin tumors, we use formalin-fixed paraffin-embedded (FFPE) biopsies of histopathologically confirmed MM, DN and CN lesions. Histopathological classification is considered the gold standard. Our long term aim is that, if achieving good results, this technique offers the possibility to be implemented in vivo in clinics and could enhance or supplement the clinical diagnosis of clinicians, avoiding the performance of biopsies in case of doubt. As a first step, we classify FFPE biopsies with our proposed methodology and compare the accuracy obtained with the accuracy achieved from in situ visual clinical inspection of dermatologists and previous studies.

In this study, 44 samples of different tumor classes (MM, DN and CN according to their histological diagnosis) were analyzed. MA methods such as Principal Component Analysis (PCA) and Partial Least Squares-Discriminant Analysis (PLS-DA) [33] were used to predict sample classification by acquiring 15–20 single Raman spectra from each tumor. The cross-validated sensitivity and specificity we obtained to distinguish DN from MM and CN are 94.1% and 100%, respectively. Furthermore, we obtained sensitivity and specificity of 82.4% and 92.9% to diagnose DN and MM samples, whose distinction is the main clinical challenge. Compared with the accuracy of GPs and dermatologists (Table S1), these results suggest that RS is a fast and non-invasive method that could be translated to clinics and implemented in vivo to enhance the clinical diagnosis of skin cancer, being especially useful for the challenging diagnostic of DN.

Additionally, we have implemented the MCR-ALS algorithm on RS images, and we were able for the first time to non-destructively localize and quantify the eumelanin pigment and the 5,6-dihydroxyindole-2-carboxylic acid (DHICA) subunit within skin lesions. This represents a step forward with respect to the currently used invasive technique HPLC. Additionally, our results suggest that the 5,6-dihydroxyindole (DHI) to DHICA ratio is higher in DN than in MM and CN lesions and, to the best of our knowledge, this is the first time this feature is reported. Furthermore, we show that this is the main feature that allows the PLS-DA method to accurately identify DN lesions. Our study points out, therefore, that the DHICA content in skin tumors should be considered as a tool to identify DN from other types of lesions.

## 2. Materials and Methods

### 2.1. Sample Preparation: Biopsy Performance, Clinical and Histopathological Examination

Skin samples were taken from normal surgical procedures during daily clinical practice and were obtained from the Hospital’s Biobank. All samples were fixed in formalin and paraffin-embedded prior to staining for histopathological diagnosis by a dermatopathologist (examples can be found in Figure S1). Unstained samples of cutaneous MM, DN and CN, were selected for RS. The age, sex and clinical and histopathological diagnoses of patients were registered (Table S2). All patients were attended at the University Hospital Virgen del Rocio de Sevilla, and this study was approved by the Research Ethics Committee of Virgen del Rocio University Hospitals, Junta de Andalucía.

### 2.2. Histological and Clinical Diagnosis

In total, 44 biopsies were classified according to their histopathological diagnosis: 14 MM, 17 DN and 13 CN. The histological classification was considered the gold standard. For each biopsy, a clinical diagnosis was also obtained. Histological and clinical diagnoses are summarized in Table S2. For a portion of samples, 10/44 biopsies, clinical and histological diagnosis differ (22.7% misclassification error). Specifically, for 1/14 (7.1%) samples of MM, 8/17 (47.1%) of DN and 1/13 (7.7%) of CN clinical and histological diagnosis are not in agreement. For DN, the percentage of misclassified samples is significantly increased, showing that DN is the most challenging tumor class to be correctly diagnosed. Additionally, for 6 out of 17 DN samples (35.3%), the clinical diagnosis was uncertain between DN and MM, reflecting that DN are difficult to be distinguished from MM lesions.

### 2.3. Raman Spectroscopy (RS): Instrumentation and Spectra Acquisition

The tissue sections analyzed by Raman spectroscopy were adjacent sections to the ones examined with histopathological methods (Figure S2). Raman spectra of non-stained tissue biopsies were obtained using a Raman microscope (inVia Renishaw, Apply Innovation, Gloucestershire, UK) that comprises a 785 nm diode laser, which was focused onto the sample via a LEICA 50× NA 0.75 objective, leading to a spatial resolution of ca. 1 µm. Single measurements were performed with a laser power of 0.15 mW, an exposure time of 10 s and a spectral resolution of 1 cm^−1^. The thickness of the tissue sections in biopsies was 4–5 µm. Since we measured in the melanotic pigmented regions, we estimated that the penetration depth of the NIR 785 nm diode laser corresponded to melanin, which was reported to be of the order of 100 µm [34]. Hence, we expect to detect signals coming from the glass substrate.

Spectral maps of 25 × 30 µm^2^ were acquired with a laser power of 0.3 mW and 2 s of acquisition time, with a spatial resolution of 1 µm and the same spectral parameters as with single measurements. These maps were later used to study the spatial distribution of molecular components (eumelanin and DHICA subunit) with the MCR-ALS algorithm.

**Figure 1:**
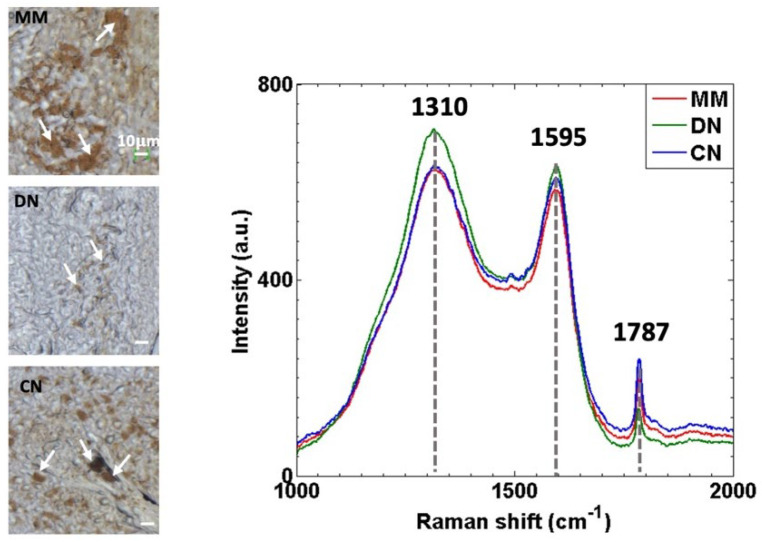
Raman spectra acquisition in the dark-pigmented regions of tumors: (**left**) Microscope bright field images of malignant melanoma (MM), dysplastic nevus (DN) and compound nevus (CN) lesions. Arrows indicate examples of the regions where Raman spectra were acquired. (**right**) Average of all Raman spectra acquired from MM (red continuous line), DN (green discontinuous line) and CN (blue dashed line). Numbers indicate the Raman shift position of bands.

For each sample, between 15–20 single Raman spectra were acquired from different dark pigmented regions of the same tumor (Figure 1a and Figure S1), corresponding to melanocytic nests where large quantities of melanin were deposited in order to capture possible pigment heterogeneity. Only tumor areas larger than 10 µm were selected for this study to ensure uniformity of the signal and avoid interference with the surrounding tissue. These measurements were later used to study the molecular content within tumors with the MCR-ALS algorithm and to classify the samples with the PLS-DA method.

On the one hand, the acquisition time was larger with single spectra than with maps because this way, the signal-to-noise ratio (S/N) was better, and, consequently, the Raman bands were more visible. On the other hand, we decided to increase the laser power and decrease the exposure time in maps because otherwise, the total acquisition time was very high (25 min/map with 2 s/pixel against 125 min/map with 10 s/pixel). Although the S/N ratio is worse in maps and the Raman bands are not so visible in their raw spectra, the total number of spectra acquired in the three maps (2250 spectra) was much higher than with single measurements (822 spectra), and it was much easier for the MCR-ALS method to accurately deconvolve the spectra into the different components. Additionally, we preferred to have a good S/N with single measurements to obtain accurate abundances of the molecular components and the accurate classification of samples.

### 2.4. Data Analysis

Before performing MA methods, preprocessing of the Raman spectra was performed in MATLAB, including the removal of cosmic ray signals and subtraction of the autofluorescence background with the method proposed in [35]. Later, Multiplicative Scatter Correction (MSC) and smoothing were performed using the PLS toolbox from Eigenvector Research. The skin tissue sections used were paraffin-embedded and placed in a glass slide for Raman measurements. The spectra obtained therefore present the background of the glass and paraffin [36,37]. In Figure S2, the spectra of the glass slide and paraffin are shown. The spectroscopic signals of the glass and paraffin were subtracted from the Raman spectra of tissues following a method based on the MCR-ALS algorithm [17]: (a) MCR-ALS algorithm was performed to the tissue spectra data set to extract a spectral component that presents the characteristic bands of the glass substrate (Figure S3). (b) The spectral component of the glass was subtracted to the dataset by: (1) multiplying the MCR loading of the glass component (blue profile on Figure S3) by the scores of this component for each Raman spectra in the data set, and (2) after, we subtracted these profiles from each spectrum in the dataset. The results are shown in Figure S4. Figure 1 shows the average of the spectra obtained for each tumor lesion after the preprocessing and background subtraction of the glass and paraffin.

Partial Least Squares-Discriminant Analysis (PLS-DA) [33] was implemented to create a model to predict and classify samples. In this case, the classification of the samples was made by introducing a priori knowledge on each sample from the histopathological examination. To perform it, only one spectrum for each sample was considered, which was the mean spectrum of all the 15–20 spectra acquired from it. Then, to assess the accuracy of the new data classification, cross-validation was applied to the data. The cross-validation method used was Venetian Blinds with 10 data splits, and values of sensitivity and specificity given by the confusion matrix were considered to evaluate its accuracy.

All Raman spectra were considered to implement the MCR-ALS algorithm. Thus, 822 spectra were included: 250 spectra corresponding to MM, 303 to DN, and 269 to CN.

Finally, Pearson’s coefficient (r) was measured to quantify the degree of spatial colocalization between different components by means of the plugging JaCoP of Fiji software [38]. For each Raman map, the score maps of the eumelanin and DHICA components obtained by the MCR were introduced in the plugging in greyscale and without setting any image threshold to consider the whole map (it can be used if it is desired to choose specific regions of the image). The values of (r) range from −1 to 1: perfect anti-colocalization (r = −1), no colocalization (r = 0), perfect colocalization (r = 1).

## 3. Results and Discussions

### 3.1. Raman Spectra of MM, DN and CN Skin Lesions Show Similar Spectral Features but Different Band Intensities

The Raman spectra of 44 tissue sections extracted from biopsies were acquired: 14 MM, 17 DN and 13 CN. The Raman spectral profile of the three tumor classes (Figure 1) showed similar bands: two broad bands centered at 1310 cm^−1^ and 1590 cm^−1^ and a narrow peak at 1787 cm^−1^. The similarity of spectral shape between different tumor classes was already described [19] and suggested that the same molecular components are present within them.

Nevertheless, subtle relative differences in their Raman band intensities can be observed. For instance, in MM and CN tumors, the intensity is lower at 1310 cm^−1^ and 1595 cm^−1^ and higher at 1787 cm^−1^ than in DN. These differences in band intensity mean that each tumor group contains different concentrations of the molecular components present in tissues and, although these appear as subtly differentiated traits by visual inspection, in this study, we show that multivariate methods such as PLS-DA or MCR-ALS are able to identify them and use them to classify skin tumors efficiently or extract meaningful molecular information, respectively. Further discussion and molecular assignment to these Raman bands are provided in the next section.

### 3.2. Raman Spectra Decomposition by Means of MCR-ALS Algorithm Enables Whole and Subunit Eumelanin Quantification in Skin Lesions

The relative differences between Raman band intensities shown in Figure 1 indicate that each lesion group (MM, DN and CN) have a different eumelanin composition. With the aim of investigating the molecular components present in skin lesions and their concentrations, Raman spectra were analyzed with the MCR-ALS algorithm. The Raman spectra of biological samples are complex because they result from the superposition of all individual spectra of each molecule present in the tissue. Thus, multivariate methods such as MCR are necessary to extract meaningful information on the molecular content of samples since this method enables us to extract the spectral profiles of the molecular components and their abundance [17,30]. The quantification performed by the MCR method is not in absolute values but in relative units, and it allows comparing the abundance of a molecular component between different samples. This means that if the score of the same component is higher in one sample than in another, its concentration in that sample is higher.

**Figure 2:**
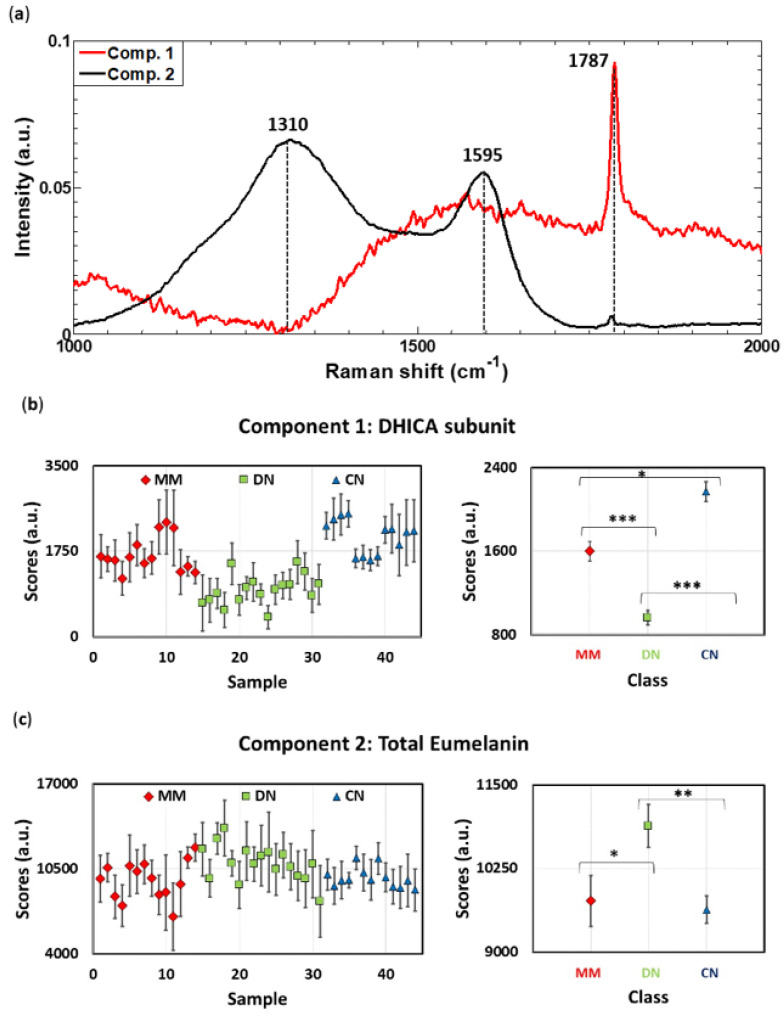
Multivariate Curve Resolution (MCR) results. Quantification of eumelanin and its 5,6−di−hydroxyindole−2−carboxylic acid (DHICA) subunit in skin tumor samples: (**a**) Component 1 is assigned to the DHICA subunit of eumelanin. Component 2 is assigned to the whole eumelanin pigment. (**b**,**c**) Left: Average MCR scores for Component 1 (**b**) and Component 2 (**c**) for each sample; vertical bars denote the standard deviation. Right: Average MCR scores for each tumor class, computed as the mean of the means shown on the left; vertical bars denote the standard error. The *p*−values associated with the t-tests for mean differences between tumor classes are shown when significant differences exist: * *p* < 0.05, ** *p* < 0.01, *** *p* < 0.001.

The Raman spectra from the skin lesion tissues were analyzed with the MCR-ALS algorithm, and two spectral components were decomposed as shown in Figure 2. Component 1 mainly consists of a sharp band at 1787 cm^−1^, also observed in Figure 1. Component 2 presents two broad bands at 1310 cm^−1^ and 1595 cm^−1^, the latter also being observed in Figure 1.

Component 2 has the same shape as the one reported for the eumelanin pigment, but with differences in the Raman peak positions of the two broad bands, previously reported to be two centered at about 1380 cm^−1^ and 1580 cm^−1^ [39]. Eumelanin, the black-to-brown pigment, is a heterogeneous polymer that consists of two subunits: 5, 6-dihydroxyindole (DHI) and 5,6-dihydroxyindole-2-carboxylic acid (DHICA). Bands at 1380 cm^−1^ and 1580 cm^−1^ are assigned to the stretching modes of C-C bonds within rings and an in-plane stretching vibration of the aromatic rings, respectively [40]. Nevertheless, differences in the peak position of this pigment were observed in different samples: sepia melanin (1400 cm^−1^, 1580 cm^−1^), synthetic melanin (1380 cm^−1^, 1590 cm^−1^), human black hair (1360 cm^−1^, 1580 cm^−1^), feline black hair (1390 cm^−1^, 1580 cm^−1^) and cutaneous melanin (1368 cm^−1^, 1572 cm^−1^) [40]. This shows that the Raman signal of melanin pigment is highly sensitive to the biochemical environment. In our case, we obtained a band at ca. 1595 cm^−1^, which corresponds to the band previously reported at 1580 cm^−1^, and the other band at ca. 1310 cm^−1^, which is shifted with respect to previous studies. This shift may be due to a different chemical environment in tumors; however, the broad glass background band at 1378 cm^−1^ probably has a bigger contribution to this shift. Despite these differences, the assignment of Component 2 to eumelanin was further corroborated by studying its spatial distribution, as shown later.

The band at 1787 cm^−1^ is barely mentioned in previous studies of eumelanin, as its intensity is usually rather low. However, it can be assigned to the carboxylic acid group of the DHICA eumelanin subunit, and it was recently considered important to estimate the DHI:DHICA ratio [41]. Therefore, Component 1 also belongs to eumelanin, but the MCR method can decompose its contribution because DHICA concentrations seem to be different in each lesion group, as inferred when considering the MCR component scores (Figure 2b).

Figure 2 also display the scores of each sample and tumor class, which reflects the abundance of each molecular component and allows relative comparisons of their concentrations. Component 2 scores, which provide an indication of the total eumelanin content [41], significantly differ between tumor classes (*p* < 0.05).

There are also significant differences in the scores of Component 1, in the order CN > MM > DN (Figure 2). This component, mainly showing the contribution of the band at 1787 cm^−1^, is assigned to the DHICA subunit of eumelanin, as it is the only subunit of this pigment with a carboxylic acid group. This, therefore, indicates that the DHICA content of DN lesions is lower than the other types of tumors, and its quantification with Raman spectroscopy should be explored as a stratification method. For future studies, it would be interesting to assign absolute values to the components obtained by implementing a regression model, calibrating it with known absolute values of pigment concentration obtained from HPLC analysis, for instance, as it was conducted with the DHI and DHICA subunits quantification in feathers [41].

### 3.3. Raman Spectroscopy Imaging Coupled with MCR-ALS Algorithm Enables Whole and Subunit Eumelanin Quantification and Localization in Skin Lesions

To gain a deeper insight into the spatial distribution of molecular components of skin lesions, we obtained Raman spectral maps of each tumor class and implemented the MCR with the acquired measurements. We obtained the same components as shown in Figure 2. The fact that any additional component was obtained from the surrounding tissue is due to a higher signal coming from the glass. Then, we obtained the scores (contributions) of the three MCR components for each pixel.

**Figure 3:**
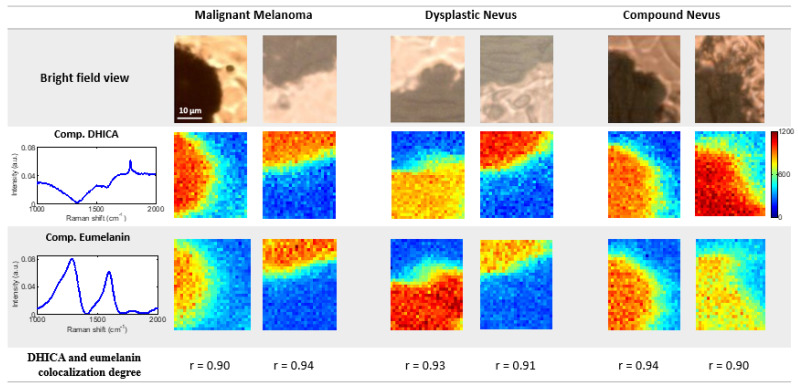
Raman spectral maps of skin lesions. Score maps of MCR components assigned to eumelanin and its DHICA subunit in samples of malignant melanoma (MM), dysplastic nevus (DN) and compound nevus (CN) are shown. From top to bottom: bright field view of the lesions; scores for Component 1 (assigned to DHICA subunit); scores for Component 2 (assigned to the whole eumelanin); degree of spatial colocalization between DHICA and eumelanin components expressed by means of Pearson’s coefficient (r), which is 1 when the colocalization is perfect. Both components are obtained only in the dark pigmented regions of lesions, supporting their assignment to the eumelanin pigment.

The spatial distribution and abundance of each component for the different skin lesions are shown in Figure 3. This shows that the scores for MCR Components 1 and 2 are higher in the dark-pigmented regions of lesions, in agreement with our assignment of these components to eumelanin, the black-to-brown pigment of the skin. Additionally, Pearson’s coefficient (r) was measured to quantify the degree of colocalization between these two components and obtained r ≥ 0.9, showing almost a perfect colocalization (r = 1) [37].

To the best of our knowledge, this is the first time that eumelanin was quantified within skin lesions non-destructively by means of RS. This is also the first time that the eumelanin DHICA content was reported as lower in DN lesions than in CN and MM lesions. This represents a step forward with respect to the currently established technique for eumelanin analysis HPLC, which is destructive and cannot provide a spatial distribution of the molecular components of lesions. Although a higher number of samples should be studied in order to establish more robust conclusions about the DHICA content in skin tumors, this parameter should be considered from now on as a tool to differentiate DN from other types of lesions.

Importantly, our results show that whole eumelanin concentration is on average higher (Figure 2c), and the DHICA concentration is lower (Figure 2b) in DN than in the other lesions. This means that the DHI:DHICA ratio is higher in DN than in other lesions. In fact, a high DHI:DHICA ratio was suggested, implying a higher cytotoxic potential of eumelanin because more cytotoxic reactive oxygen species (ROS) are generated during the production of the DHI subunit than during the production of the DHICA subunit in the biosynthesis of eumelanin in melanocytes [41]. Therefore, this higher DHI:DHICA ratio may explain the known susceptibility of DN to develop into malignant melanomas due to the altered regulation of oxidative stress [42,43]. The relatively high intramelanocytic oxidative stress that a high DHI:DHICA ratio in eumelanin pigments can create was also proposed to explain the low survival of melanocytes with no activity of the enzymes Tyrp1 and Tyrp2, involved in the synthesis of the DHICA subunit [44]. The high DHI:DHICA ratio in DN found here, consequently, not only represents a novel tool to non-invasively identify lesions with a risk of becoming MM but also a possible strategy against melanoma if mechanisms to avoid oxidative stress related to the high DHI:DHICA ratio are considered.

### 3.4. Raman Spectroscopy Coupled with PLS-DA Efficiently Classifies MM, DN and CN Skin Lesions

In the previous sections, we studied the eumelanin content within skin tumors and observed that there are significant differences in the DHICA content of DN lesions, being higher in MM and CN. In order to exploit this property, PLS-DA models were built to distinguish DN lesions from the rest. For this, the class assignment (tumor type) was based on the histopathological assessment of lesions.

In Table 1, cross-validated sensitivity and specificity values of three different models are depicted. Model 1 distinguishes DN lesions from MM and CN, considering the last two as a single class. Model 2 classifies DN and MM lesions uniquely, and Model 3 classifies DN and CN lesions.

Considering Model 1, sensitivity and specificity of 94.1% and 100%, respectively, were obtained for DN class. This is a promising result since DN was always considered to be intermediate between MM and CN. Additionally, the VIP (Variable Importance of Projection) scores [45] of the model were computed, as they show which are the most important bands while classifying samples. As shown in Figure S5, the most relevant band is the one at 1787 cm^−1^, belonging to the DHICA eumelanin subunit. That is, this outlines the fact that the difference in the DHICA content allows an efficient diagnosis of DN lesions by means of RS.

Model 2 is especially relevant as the distinction between DN and MM is clinically challenging. In this model, we obtained sensitivity and specificity of 82.4% and 92.9% for the MM class. Overall, the accuracy is higher than the previous methods (Table S1). Annessi et al. [32] compared the three most common methods of dermoscopic diagnosis, obtaining 78.1–85.4% sensitivities and 64.7–79.4% specificities; and Zhang and Li [5] implemented a method based on the immunochemical detection of four biomarkers, with 94.3% sensitivity and 81.2% specificity. The sensitivity obtained in this last study was very high, but the proposed method is invasive, time-consuming and complex.

Model 3 show 100% sensitivity and specificity while diagnosing DN and CN lesions. Better accuracy of this model than Model 2 can be argued by the fact that bigger differences were obtained on average in the DHICA content between DN-CN than between DN mm (Figure 2b).

We would like to stress that most DN lesions were distinguished from MM and CN lesions by detecting a higher DHI:DHICA ratio in this lesion group. This suggests that the DHI:DHICA ratio may be an indicator of the tumoral behavior in skin pigmented lesions and, to the best of our knowledge, little emphasis was placed on the estimation of this ratio. In fact, in recent decades, all the attention was focused on estimating the pheomelanin:eumelanin ratio, as it is generally suggested that a high ratio increases cancer risk and malignancy [46]. However, considering the results presented in this paper, it seems that more efforts should be made to study the DHI and DHICA content in skin lesions. In addition, in this study, MM and DN lesions were classified independently on their grade of malignancy or dysplasia, respectively, but, as can be seen in Figure 2, there is intraclass variability regarding the whole eumelanin and DHICA content. For future research, it will be interesting to consider the different grades of malignancy and dysplasia in MM and DN, respectively, and study if they are correlated with their DHI:DHICA ratio.

## 4. Conclusions

In this paper, we show, for the first time, a novel methodology based on RS and coupled with MCR-ALS able to localize and quantify the distribution of the total eumelanin and the DHICA subunit content within skin lesions non-destructively. This represents a step forward with respect to the currently established technique for melanin analysis, HPLC, which is invasive, destructive and cannot provide spatial information about molecular components. We also report for the first time that the eumelanin DHICA content is lower in DN lesions than in CN and MM lesions, a property used by the PLS-DA method to identify DN lesions with classification sensitivity and specificity of 94.1% and 100%, respectively.

A high DHI:DHICA ratio was previously suggested to imply a higher cytotoxic potential of eumelanin [41]. Therefore, the higher DHI:DHICA ratio observed in DN may explain its susceptibility to develop into malignant melanomas due to the altered regulation of oxidative stress. Thus, the high DHI:DHICA ratio in DN may represent a novel tool to non-invasively identify lesions with a risk of becoming MM and a possible strategy against melanoma by means of mechanisms that prevent oxidative stress related to the high DHI:DHICA ratio. Nonetheless, a larger number of samples should now be studied in order to better assess the accuracy of this methodology for routine use in vivo in clinics.

## Figures and Tables

**Table 1:** Cross-validated sensitivity and specificity values of three PLS-DA models that distinguish dysplastic nevi (DN, *n* = 17) from malignant melanomas (MM, *n* = 14) and compound nevi (CN, *n* = 13). Model 1 distinguishes DN class from MM and CN classes considered together, Model 2 distinguishes DN from MM and Model 3 distinguishes DN from CN.

**Model 1: DN vs. [MM and CN]**		
	DN	MM and CN
Sensitivity (%)	94.1	100
Specificity (%)	100	94.1
**Model 2: DN vs. MM**		
	DN	MM
Sensitivity (%)	82.4	92.9
Specificity (%)	92.9	82.4
**Model 3: DN vs. CN**		
	DN	CN
Sensitivity (%)	100	100
Specificity (%)	100	100

## Data Availability

Data are contained within the article or Supplementary Materials.

## Supplementary Materials

The following are available online at https://www.mdpi.com/article/10.3390/cancers14041056/s1, Figure S1: Examples of images of tissue sections H&E stained, Figure S2: Raman spectra obtained from the glass substrate, Figure S3: Loadings of the MCR-ALS components used for background subtraction, Figure S4: Glass and paraffin background subtraction figures, Figure S5: Variable Importance of Projection (VIP) scores of the PLS-DA Model 1 shown in Table 1. Table S1: Sensitivity and specificity values of previous proposed diagnostic methods to classify malignant melanomas (MM), dysplastic nevi (DN) and compound nevi (CN), Table S2: Clinical data of the skin tumors analyzed in this study. References in Supplementary Material are cited in main text as: [5,14,19,21,24,26,32,45].
