# Supplementary material for: Novel Non-Invasive Quantification and Imaging of Eumelanin and DHICA Subunit in Skin Lesions by Raman Spectroscopy and MCR Algorithm: Improving Dysplastic Nevi Diagnosis
Source: Cancers (Basel). 2022 Feb 18;14(4):1056. doi: 10.3390/cancers14041056 (PMC8870175; doi:10.3390/cancers14041056)
Supplement: Supplementary file 1 [file cancers-14-01056-s001.zip › cancers-1524189-supplementary.pdf]

cancers

MDPI

Supplementary Material

Novel Non-Invasive Quantification and Imaging of Eumelanin and DHICA Subunit in Skin Lesions by Raman Spectroscopy and MCR Algorithm: Improving Dysplastic Nevi Diagnosis

José Javier Ruiz [1,†], Monica Marro [1,†,*], Ismael Galván [2], José Bernabeu-Wittel [3], Julián Conejo-Mir [3], Teresa Zulueta-Dorado [3], Ana Belén Guisado-Gil [3] and Pablo Loza-Álvarez [1,*]

[1] ICFO-Institut de Ciencies Fotoniques, The Barcelona Institute of Science and Technology, 08860 Castelldefels, Spain; jose-javier.ruiz@icfo.eu
[2] Department of Evolutionary Ecology, National Museum of Natural Sciences, CSIC, 28006 Madrid, Spain; galvan@mncn.csic.es
[3] Department of Dermatology, University Hospital Virgen del Rocio,41013 Sevilla, Spain; jose.bernabeu.sspa@juntadeandalucia.es (J.B.-W.); jsconejomir@us.es (J.C.-M.); teresa.zulueta.sspa@juntadeandalucia.es (T.Z.-D.); anab.guisado.sspa@juntadeandalucia.es (A.B.G.-G.)
* Correspondence: monica.marro@icfo.eu (M.M.); pablo.loza@icfo.eu (P.L.-Á.)
† These authors contributed equally to this paper.

Accuracy of previous proposed methods in skin cancer diagnosis

Table S1. Sensitivity and specificity values of previous proposed diagnostic methods to classify malignant melanomas (MM), dysplastic nevi (DN) and compound nevi (CN).

Study	Skin Lesions Analysed	Classification	Method	Sensitivity (%)	Specificity (%)
Menzies at al. (2005) [1]	2340 lesions (382 MM, 602 DN, 207 CN, etc.)	MM vs. other malignant and benign lesions (DN and CN among others)	Automated instrument called SolarScan based on image analysis of dermoscopy (surface microscopy) features	85	65
			Dermoscopy experts	90	59
			Dermatologists	81	60
			Trainees dermatologists	85	36
			General practitioners	62	63
Annessi et al. (2007) [2]	198 atypical macular melanocytic lesions (98 MM and 102 DN)	MM vs. DN	Pattern analysis	85.4	79.4
Lui et al. (2012) [3]	518 validated lesions from 453 subjects	44 MM vs. 286 non-melanoma pigmented skin lesions (DN and CN among others)	Real-time and in vivo Raman Spectroscopy together with PCA-GDA and PL analyses	90-99	15-68
Zhang and Li (2012) [4]	155 skin lesions (122 MM and 33 DN)	MM vs. DN	Combining the immunohistochemical expression of 4 biomarkers	94.3	81.2

Santos et al. (2018) [5]	174 skin lesions (91 MM, 44 DN, 43 CN, etc.)	MM vs common nevi (CN, dermal, intradermal, junctional and blue nevi)	Raman spectroscopy performed in ex vivo samples combined with PCA-LDA	100	43.8
			DN samples were excluded before calibrating the predictive model. Later, they were classified	Of 44 DN samples, 34 were classified as MM (73.9 %) and 12 were classified as CN (26.1 %)	
Feng et al. (2018) [6]	12 MM and 17 DN lesions	MM vs. DN	Implementing an inverse biophysical model of skin components obtained in situ with Raman Spectroscopy and logistic regression classifiers	95	94
Zhao et al. (2019) [7]	731 validated lesions from 644 patients (60 MM, 85 DN, 32 CN, etc.)	Cancerous/precancerous (MM among others) vs benign cases (DN and CN among others)	Real-time and in vivo Raman Spectroscopy together with PCA-GDA and PL analyses and including patient demographic information (gender, skin type, lesion location, age)	90	80.8

Sample data

Table S2. Clinical data of the skin tumors analyzed in this study. From left to right: sample number, sex and age of patients, clinical diagnosis and histological diagnosis of malignant melanomas (MM), dysplastic nevi (DN) and compound nevi (CN). Clinical diagnosis is the one performed by visual inspection of the skin lesion and histological diagnosis is the one performed through histological analysis of the biopsy, and it has been considered as gold standard.

Sample	Sex (Male/Female)	Age (Years)	Clinical Diagnosis	Histological Diagnosis
**1**	M	49	MM	MM
**2**	F	66	MM	MM
**3**	M	85	MM	MM
**4**	F	77	MM	MM
**5**	F	71	MM	MM
**6**	F	23	MM	MM
**7**	M	93	MM	MM
**8**	M	63	MM	MM
**9**	F	72	Not Available	MM
**10**	F	44	MM	MM
**11**	M	79	MM	MM
**12**	F	32	MM	MM
**13**	M	39	MM	MM
**14**	M	66	MM	MM
**15**	M	49	CN	DN
**16**	F	49	DN or MM	DN
**17**	M	40	DN	DN
**18**	M	30	DN or MM	DN
**19**	M	26	DN or MM	DN
**20**	M	38	DN or MM	DN

**21**	F	44	DN or MM	DN
**22**	M	36	DN	DN
**23**	F	26	DN	DN
**24**	F	70	Lentigo	DN
**25**	M	63	DN or MM	DN
**26**	F	32	DN	DN
**27**	M	30	DN	DN
**28**	M	38	DN	DN
**29**	M	28	DN	DN
**30**	M	20	DN	DN
**31**	F	21	DN	DN
**32**	F	13	CN	CN
**33**	M	43	DN or MM	CN
**34**	F	39	CN	CN
**35**	F	18	CN	CN
**36**	F	27	CN	CN
**37**	M	47	CN	CN
**38**	F	22	CN	CN
**39**	F	71	CN	CN
**40**	F	32	CN	CN
**41**	M	64	CN	CN
**42**	M	54	CN	CN
**43**	F	36	CN	CN
**44**	F	37	CN	CN

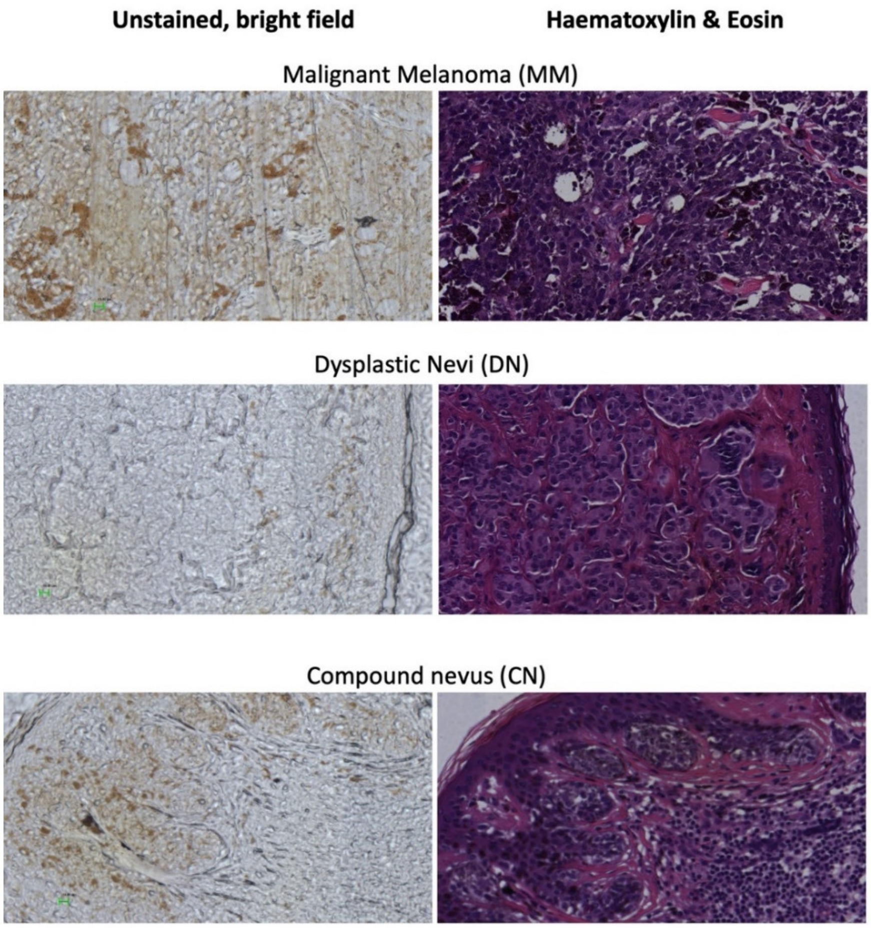

Figure S1. Examples of tissue images for each skin lesion type. Consecutive tissue sections were used: unstained tissues (bright field on the left), and its corresponding Hematoxylin & eosin-stained tissue (right). Green scale bar is 10µm.

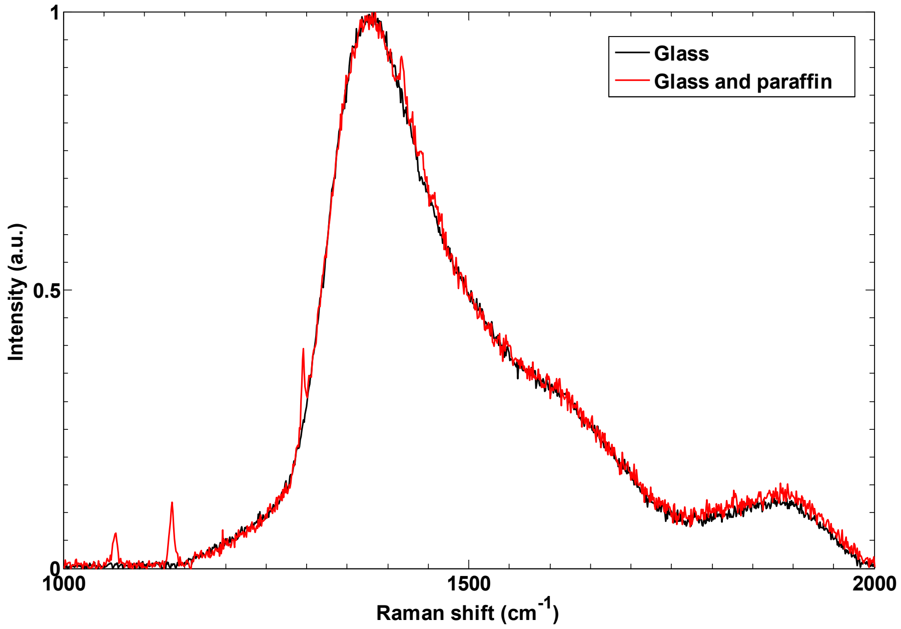

Figure S2. Raman spectra obtained from the glass substrate (black) and the Glass substrate in the area where paraffin is present (red).

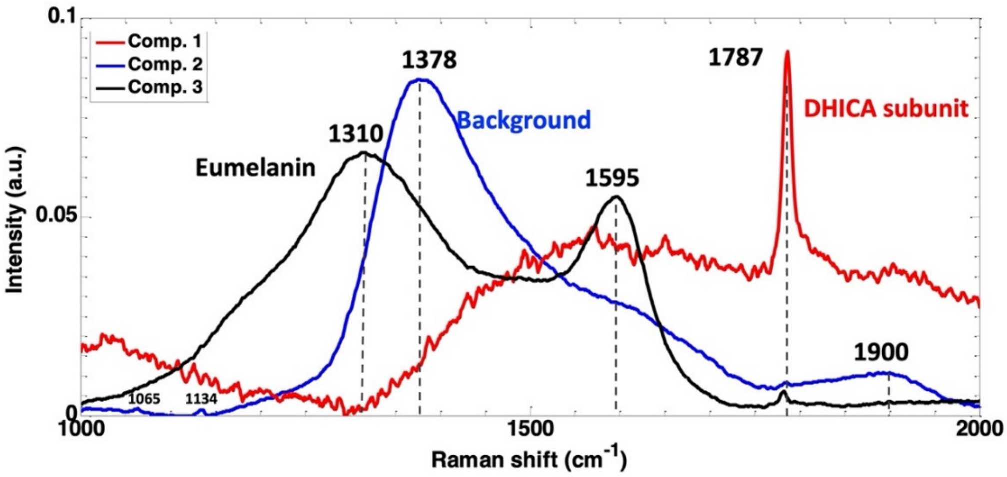

Figure S3. Loadings of the MCR-ALS components obtained with the pre-processed Raman spectra of tissues and including 20 spectra of the glass substrate to extract the background signal of the substrate and paraffin in the data. Component 2 has the characteristic bands of the glass and paraffin. This component will be subtracted to the pre-processed data to subtract the background (see Figure S4).

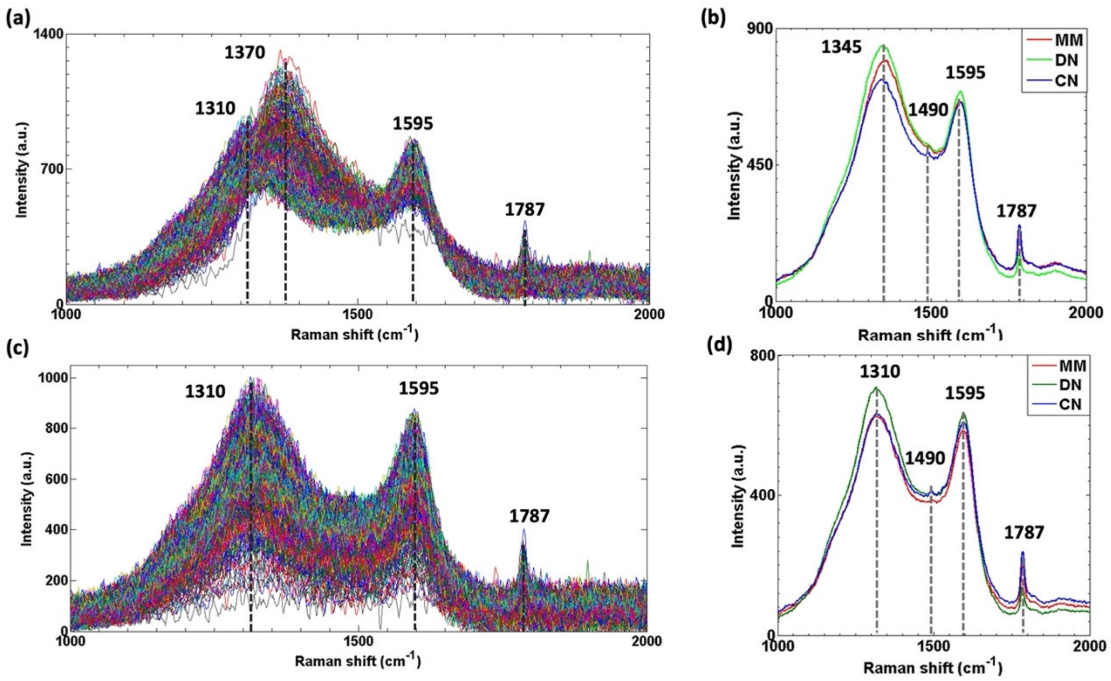

Figure S4. Glass and paraffin background subtraction: (**a**) Pre-processed raw Raman spectra. (**b**) Average of the spectra for each skin tissue lesion for the spectra in (**a**). (**c**) Tissue spectra after background subtraction by means of the MCR-ALS background component (Figure. S2). (**d**) Average of the spectra for each skin tissue lesion for the spectra in (**b**).

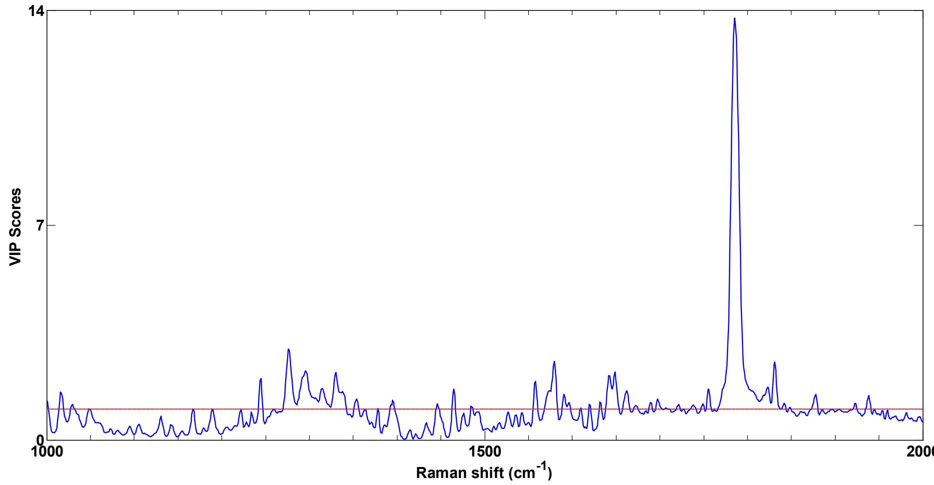

Figure S5: Variable Importance of Projection (VIP) scores [8] of the PLS-DA Model 1 shown in Table 1. These scores express the importance of each Raman shift variable while diagnosing DN lesions against MM and CN ones. The most important band is the one at 1787 cm^{-1}, assigned to the carboxylic acid group of the DHICA subunit of the eumelanin pigment.

References

1. Menzies, S.W.; Bischof, L.; Talbot, H.; Gutenev, A.; Avramidis, M.; Wong, L.; Lo, S.K.; Mackellar, G.; Skladnev, V.; McCarthy, W.; et al. The performance of SolarScan: An automated dermoscopy image analysis instrument for the diagnosis of primary melanoma. *Arch Dermatol*. **2005**, *141*, 1388–1396; Correction in *Arch Dermatol*. **2006**, *142*, 558. https://doi.org/10.1001/archderm.141.11.1388.
2. Annessi, G.; Bono, R.; Sampogna, F.; Faraggiana, T.; Abeni, D. Sensitivity, specificity, and diagnostic accuracy of three dermoscopic algorithmic methods in the diagnosis of doubtful melanocytic lesions: The importance of light brown structureless areas in differentiating atypical melanocytic nevi from thin melanomas. *J. Am. Acad. Dermatol*. **2007**, *56*, 759–767. https://doi.org/10.1016/j.jaad.2007.01.014.
3. Lui, H.; Zhao, J.; McLean, D.; Zeng, H. Real-time Raman spectroscopy for in vivo skin cancer diagnosis. *Cancer Res*. **2012**, *72*, 2491–2500. https://doi.org/10.1158/0008-5472.CAN-11-4061.

4. Zhang, G.; Li, G. Novel multiple markers to distinguish melanoma from dysplastic nevi. *PLoS ONE* **2012**, *7*, e45037. https://doi.org/10.1371/journal.pone.0045037.
5. Santos, I.P.; Van Doorn, R.; Caspers, P.J.; Schut, T.C.B.; Barroso, E.; Nijsten, T.E.C.; Hegt, V.N.; Koljenović, S.; Puppels, G.J. Improving clinical diagnosis of early-stage cutaneous melanoma based on Raman spectroscopy. *Br. J. Cancer* **2018**, *119*, 1339–1346. https://doi.org/10.1038/s41416-018-0257-9.
6. Feng, X.; Moy, A.J.; Nguyen, H.; Zhang, Y.; Zhang, J.; Fox, M.C.; Sebastian, K.R.; Reichenberg, J.S.; Markey, M.K.; Tunnell, J.W. Raman biophysical markers in skin cancer diagnosis. *J. Biomed. Opt*. **2018**, *23*, 057002.
7. Zhao, J.; Zeng, H.; Kalia, S.; Lui, H. Incorporating patient demographics into Raman spectroscopy algorithm improves in vivo skin cancer diagnostic specificity. *Transl. Biophotonics* **2019**, *1*, e201900016. https://doi.org/10.1002/tbio.201900016.
8. Chong, I.; Jun, C. Performance of some variable selection methods when multicollinearity is present. *Chemom. Intell. Lab. Syst.* **2005**, *78*, 103–112. https://doi.org/10.1016/J.CHEMOLAB.2004.12.011.
